# Point-of-Care Assessment of Kidney Function in Chronic Kidney Disease: Current Technologies and Future Perspectives

**DOI:** 10.3390/medsci14050521

**Published:** 2026-08-27

**Authors:** Atthaphong Phongphithakchai, Kraiyasak Wongna, Ratana Netphakdee, Wiyada Kwanhian Klangbud, Jongkonnee Thanasai, Fumitaka Kawakami, Moragot Chatatikun

**Affiliations:** 1 Nephrology Unit, Division of Internal Medicine, Faculty of Medicine, Prince of Songkla University, Songkhla 90110, Thailand; atthaphong.p@psu.ac.th; 2 School of Allied Health Sciences, Walailak University, Nakhon Si Thammarat 80160, Thailand; kaiyasak.wo@mail.wu.ac.th (K.W.); ratana.ne@mail.wu.ac.th (R.N.); 3 Biomedical Sciences and Health Innovations (International Program), School of Allied Health Sciences, Walailak University, Nakhon Si Thammarat 80160, Thailand; 4 Medical Technology Program, Faculty of Science, Nakhon Phanom University, Nakhon Phanom 48000, Thailand; wiyadakwanhian@gmail.com; 5 Faculty of Medicine, Mahasarakham University, Mahasarakham 44000, Thailand; jongkonnee@msu.ac.th; 6 Department of Regulation Biochemistry, Graduate School of Medical Sciences, Kitasato University, Sagamihara 252-0373, Japan; kawakami@kitasato-u.ac.jp; 7 Research Excellence Center for Innovation and Health Products, Walailak University, Tha Sala, Nakhon Si Thammarat 80161, Thailand

**Keywords:** chronic kidney disease, point-of-care testing, kidney function, biomarkers

## Abstract

Chronic kidney disease (CKD) requires assessment of both glomerular filtration and kidney damage, particularly albuminuria, for diagnosis, risk stratification, and longitudinal care. Point-of-care testing (POCT) can shorten turnaround time and improve access when conventional laboratory testing is unavailable or would delay a clinical pathway. This narrative review summarizes established and emerging POCT approaches for kidney assessment in CKD, with emphasis on creatinine/eGFR and urine albumin-to-creatinine ratio (UACR), and distinguishes analytical validity from workflow utility and patient-level clinical utility. Evidence is strongest for rapid creatinine measurement in selected workflows, especially pre-imaging assessment and decentralized screening, while quantitative/semiquantitative UACR POCT can support CKD detection when abnormal results are appropriately confirmed. Cystatin C microfluidic systems remain investigational, and kidney injury/stress or molecular biomarkers such as NGAL, KIM-1, L-FABP, [TIMP-2]·[IGFBP7], extracellular vesicles, and microRNAs should be regarded primarily as a research horizon rather than direct measures of GFR. Important implementation requirements include assay-specific validation, calibration traceability, quality assurance, recognition of biological and analytical interference, and confirmation of results near major clinical decision thresholds. Evidence that POCT improves long-term CKD outcomes, safety, adherence, or cost-effectiveness remains limited. Future studies should prioritize prospective clinical utility, implementation, cost-effectiveness, home-use validation, and patient-centered outcomes.

## 1. Introduction

Chronic kidney disease (CKD) has become one of the fastest-growing causes of morbidity and mortality worldwide and is projected to remain a major contributor to global disability over the coming decades. Because disease progression is often clinically silent, timely assessment of kidney function is essential for early diagnosis, risk stratification, and treatment optimization [1,2,3]. Assessment of kidney function is central to the diagnosis, staging, prognosis, and management of CKD. Current international guidelines recommend serum creatinine-based estimated glomerular filtration rate (eGFR) together with urine albumin-to-creatinine ratio (UACR) for CKD detection and risk stratification. These measurements guide clinical decision-making, including medication dose adjustment, referral to nephrology services, prediction of disease progression, and preparation for kidney replacement therapy [4].

Despite their widespread use, conventional laboratory-based kidney function tests have several practical limitations. Samples must be transported to centralized laboratories, processed using automated analyzers, and reported back to clinicians, resulting in turnaround times that may range from several hours to days depending on healthcare infrastructure. Such delays may postpone clinical decisions in outpatient clinics, primary care, emergency departments, rural healthcare facilities, and community CKD screening programs. Repeated laboratory visits also increase healthcare costs and may reduce patient adherence to long-term monitoring [5].

Point-of-care testing (POCT) has emerged as a promising strategy to overcome these limitations by providing rapid biochemical measurements close to the patient [6]. Handheld and portable analyzers are now capable of measuring serum creatinine within minutes using small volumes of capillary or venous blood, with many systems automatically calculating eGFR. Rapid availability of kidney function results may facilitate timely clinical decision-making, improve access to CKD screening, support medication safety before exposure to nephrotoxic agents or contrast media, and expand kidney care in resource-limited settings. The Kidney Disease: Improving Global Outcomes (KDIGO) 2024 clinical practice guideline for the evaluation and management of chronic kidney disease recommends consideration of point-of-care creatinine and urine albumin measurements when laboratory access is limited or when POCT facilitates the clinical pathway, provided that quality assurance systems are in place [4,7].

Technological advances have further accelerated the development of kidney function POCT. Commercial devices such as StatSensor^®^, i-STAT^®^, epoc^®^, and ABL analyzers are increasingly used in clinical practice, while emerging technologies including electrochemical biosensors, microfluidic platforms, paper-based diagnostics, nanomaterial-enabled sensors, and smartphone-integrated systems are expanding the possibilities for rapid kidney function assessment [6,8]. In parallel, novel biomarkers including cystatin C, neutrophil gelatinase-associated lipocalin (NGAL) and other biosensor-compatible analytes are being investigated as potential next-generation point-of-care markers capable of providing complementary information beyond serum creatinine alone [9,10].

Although numerous point-of-care platforms have been developed, the evidence base spans different levels of maturity. In this review, “kidney assessment” is used as the broader framework: creatinine, cystatin C, and eGFR are filtration measures; UACR reflects kidney damage and is essential to CKD classification; and injury/stress or molecular biomarkers provide complementary biological information but are not direct measures of GFR. The review therefore emphasizes established POCT for creatinine and albuminuria, while retaining emerging biomarkers in a deliberately concise research-horizon section. Throughout, analytical validity, clinical validity, workflow utility, and patient-level clinical utility are distinguished to avoid extrapolating technical feasibility into proven clinical benefit.

## 2. Review Methodology

This narrative review was informed by literature searches of PubMed/MEDLINE, Embase, and Google Scholar, supplemented by KDIGO guidance, regulatory/manufacturer documentation, and reference-list screening. Searches covered database inception through 1 July 2026 and combined terms related to chronic kidney disease, point-of-care testing, creatinine, eGFR, albuminuria/UACR, cystatin C, handheld or portable analyzers, biosensors, microfluidics, kidney injury biomarkers, telemedicine, and home monitoring. English-language human studies, analytical validation studies, systematic reviews, guidelines, and primary technology-validation reports were prioritized. For established devices, studies reporting method comparison, bias/imprecision, diagnostic classification, sample requirements, or workflow outcomes were preferentially selected. For emerging biomarkers, claims of POCT readiness were retained only when supported by a device-development or analytical-validation study in biological samples; otherwise, the technology was classified as research-stage. Because this was a narrative rather than systematic review, no formal risk-of-bias meta-analysis was performed. All citations were re-audited against the specific statements they support during revision.

## 3. Clinical Framework for Kidney Assessment

Assessment of kidney function is fundamental to the diagnosis, staging, and longitudinal monitoring of CKD. An ideal marker of kidney function should accurately reflect GFR, respond rapidly to changes in renal function, be minimally affected by non-renal factors, and be readily measurable using a rapid, inexpensive, and reproducible assay. From the perspective of POCT, additional characteristics including portability, minimal sample volume, short turnaround time, and ease of operation are equally important to facilitate decentralized healthcare delivery and community-based kidney disease screening [4].

### 3.1. Serum Creatinine

Serum creatinine remains the most widely used endogenous biomarker for assessing kidney function because of its low cost, broad availability, and extensive clinical validation. Creatinine is generated from skeletal muscle creatine metabolism and is eliminated primarily by glomerular filtration, although approximately 10–20% is secreted by the proximal renal tubules. Consequently, serum creatinine concentration is influenced not only by GFR but also by age, sex, ethnicity, muscle mass, dietary meat intake, medications, and tubular secretion, limiting its sensitivity for detecting early kidney dysfunction [11,12]. Despite these limitations, serum creatinine remains the cornerstone of routine kidney function assessment because it is standardized against isotope dilution mass spectrometry (IDMS), enabling consistent estimation of GFR across laboratories [13]. Advances in assay standardization have substantially improved inter-laboratory agreement and facilitated widespread implementation of creatinine-based eGFR equations in clinical practice.

### 3.2. Cystatin C

Cystatin C has emerged as an important alternative endogenous filtration marker because it is produced at a relatively constant rate by all nucleated cells, freely filtered by the glomerulus, and almost completely reabsorbed and metabolized within proximal tubular cells without significant tubular secretion. Compared with serum creatinine, cystatin C is less affected by muscle mass, sex, diet, and frailty, making it particularly valuable in older adults, individuals with sarcopenia, malnutrition, obesity, or liver disease [14].

### 3.3. Estimated Glomerular Filtration Rate

Because serum creatinine alone does not directly reflect filtration capacity, eGFR is used for CKD staging and risk assessment. The 2021 CKD-EPI creatinine equation is race-free and is incorporated into contemporary CKD guidance [4]. However, eGFR is an estimate and its interpretation depends on clinical context. Creatinine-based eGFR equations assume approximate steady state and can be misleading during rapidly changing kidney function, including evolving AKI. For medication dosing, indexed eGFR (mL/min/1.73 m^2^), body-surface-area–deindexed or absolute GFR (mL/min), and creatinine clearance are not universally interchangeable; clinicians should use the renal-function metric specified in the relevant drug label or validated dosing evidence. In addition, eGFR equations displayed by POCT systems are device-, software-, and jurisdiction-dependent rather than uniform across all analyzers. Equations combining creatinine and cystatin C generally improve GFR estimation when creatinine alone is unreliable [4,15].

### 3.4. Characteristics of an Ideal Biomarker for Point-of-Care Kidney Function Assessment

Although serum creatinine remains the current standard for POCT, the ideal kidney function biomarker has not yet been identified. From both analytical and clinical perspectives, an optimal POCT biomarker should accurately reflect true GFR across the entire spectrum of kidney function, demonstrate high analytical precision and reproducibility, remain minimally affected by physiological or pathological confounders, and rapidly detect clinically meaningful changes in renal function [6]. Equally important, POCT implementation requires biomarkers that can be measured using portable, inexpensive devices requiring only a small volume of whole blood with minimal operator training and rapid turnaround times. As biosensor technologies continue to evolve, emerging biomarkers such as cystatin C and other filtration markers may overcome several limitations of creatinine-based assessment and further improve the accuracy and accessibility of decentralized function testing.

## 4. Established Point-of-Care Technologies

Point-of-care kidney function testing encompasses a heterogeneous group of technologies ranging from single-use handheld electrochemical systems to compact benchtop chemistry and blood gas analyzers. Serum or whole-blood creatinine remains the principal analyte measured by currently available platforms because it is inexpensive, familiar to clinicians, and directly compatible with established eGFR equations. However, individual systems differ substantially in sample requirements, analytical principle, turnaround time, calibration, susceptibility to interference, and agreement with central laboratory methods. These differences are clinically important because even modest creatinine bias may shift a patient across an eGFR threshold used for CKD classification, medication dosing, contrast-enhanced imaging, or nephrology referral [13,16,17]. In this review, “commercially available” denotes products marketed in at least one jurisdiction as of August 2026; regulatory clearance, intended use, cartridges, eGFR software, and quality-control requirements vary by country and device generation and should be verified from local manufacturer/regulatory documentation.

### 4.1. Point-of-Care Creatinine Testing

Point-of-care creatinine testing is currently available through a variety of handheld and benchtop platforms that differ in analytical principle, sample type, turnaround time, and clinical performance [17,18,19,20,21,22,23,24,25,26,27,28]. The major commercially available creatinine POCT platforms and their key characteristics are summarized in Table 1.

#### 4.1.1. Analytical Principles

Conventional laboratory creatinine measurement is principally based on either the alkaline picrate, or Jaffe, reaction or enzymatic assays. The Jaffe method is inexpensive and widely available but lacks complete analytical specificity because non-creatinine chromogens including glucose, ketones, proteins, bilirubin, and some medications may produce positive or negative interference. Enzymatic laboratory assays generally offer improved specificity, although they are more expensive and remain vulnerable to selected analytical interferences [11,15]. Most handheld creatinine POCT systems use enzymatic electrochemical methods. Creatinine is converted through a sequence of enzyme-mediated reactions, commonly involving creatininase, creatinase, and sarcosine oxidase. The resulting electroactive product, typically hydrogen peroxide, is detected at an electrode, and the generated current is proportional to the creatinine concentration. This approach is often described as amperometric detection, which is a subtype of electrochemical measurement. Thus, “enzymatic,” “electrochemical,” and “amperometric” describe related rather than mutually exclusive components of the assay: enzymatic reactions provide molecular recognition, whereas amperometry converts the biochemical reaction into a measurable electrical signal [16,17,18]. Compact chemistry analyzers such as Piccolo Xpress use a different format. Creatinine is measured through coupled enzymatic reactions followed by photometric detection in a disposable reagent disc. These systems require more sample and are less portable than handheld meters, but they can simultaneously measure creatinine, BUN, electrolytes, and other biochemical parameters [19,20].

#### 4.1.2. Nova StatSensor Creatinine/eGFR

The StatSensor Creatinine/eGFR system (Nova Biomedical, Waltham, MA, USA) is a handheld device that measures creatinine using a disposable electrochemical biosensor. It accepts capillary, venous, or arterial whole blood and requires a very small sample volume, making finger-stick testing feasible. Creatinine and calculated eGFR can be displayed rapidly, supporting use in community screening, radiology departments, outpatient clinics, and settings where venous phlebotomy or immediate laboratory access is unavailable [6,21]. StatSensor offers several practical advantages. The system is compact, requires only microliter-scale blood volume, and has a relatively simple operating procedure. These characteristics make it attractive for decentralized testing and screening by appropriately trained non-laboratory personnel [21]. Its portability may be particularly advantageous in geographically isolated populations and in patients who require kidney function assessment immediately before contrast-enhanced imaging. Nevertheless, the performance of StatSensor has not been uniform across studies. Evaluations have reported acceptable correlation with central laboratory creatinine, but correlation alone does not establish clinical interchangeability [22,23]. Proportional bias has been observed, particularly at higher creatinine concentrations, and performance may deteriorate in patients with reduced kidney function. In a study that included patients with CKD and kidney transplant recipients, StatSensor demonstrated clinically relevant bias and did not consistently satisfy analytical performance goals across the measured range [22]. A community-based comparison also found that StatSensor performed reasonably at low creatinine concentrations but showed proportional bias over a wider concentration range, whereas i-STAT showed closer agreement with the laboratory comparator [23]. These findings are particularly relevant to CKD because errors at high creatinine concentrations may substantially alter eGFR classification. A device that performs adequately for excluding severe renal impairment before imaging may therefore not necessarily be suitable for longitudinal monitoring of advanced CKD. Local validation against an IDMS-traceable laboratory assay and confirmation of unexpected or threshold-adjacent results remain essential.

#### 4.1.3. Abbott i-STAT

The i-STAT system (Abbott Point of Care Inc., Princeton, NJ, USA) is a handheld cartridge-based analyzer capable of measuring creatinine alongside selected blood gases, electrolytes, and metabolites. Creatinine is measured in whole blood by an enzymatic amperometric method. The portability of the analyzer and disposable cartridge format allow results to be produced near the patient from a small sample, while electronic connectivity facilitates transfer to clinical information systems [16,24]. Among commonly evaluated creatinine POCT devices, i-STAT has generally demonstrated relatively favorable analytical performance. A systematic review and economic evaluation of POCT before contrast-enhanced computed tomography found that i-STAT and ABL-based systems were more accurate than StatSensor for detecting patients below clinically important eGFR thresholds, although the quantity and quality of evidence varied among devices and patient groups [17]. In community CKD testing, i-STAT showed strong correlation and small mean bias compared with reference laboratory creatinine, without clear evidence of proportional bias in the evaluated population [23]. However, acceptable average agreement does not eliminate patient-level error. Bias may vary according to specimen type, creatinine concentration, calibration lot, environmental conditions, and comparator method [24]. Cartridge-based systems also entail ongoing consumable costs and require controlled storage, operator training, quality-control procedures, and connectivity governance. Therefore, i-STAT results should not automatically be considered interchangeable with central laboratory measurements, especially when small changes are used to judge CKD progression.

#### 4.1.4. Siemens Epoc Blood Analysis System

The epoc system (Siemens Healthineers, Erlangen, Germany) is a portable wireless blood-analysis platform that measures blood gases, electrolytes, metabolites, and creatinine using a single-use test card. Creatinine is determined using enzymatic amperometry in heparinized or non-anticoagulated arterial, venous, or capillary whole blood [17,25]. The capacity to measure multiple analytes from one specimen is advantageous in emergency, perioperative, and intensive care environments, particularly when kidney function must be interpreted together with potassium, acid–base status, glucose, and lactate. Nevertheless, creatinine has been one of the more analytically challenging analytes on the epoc platform. In an evaluation of all analytes available on the system, creatinine demonstrated greater bias than other measurements when compared with central laboratory methods. Specifically, epoc creatinine showed a mean bias of approximately +13.5% versus the Abbott Architect Jaffe method and +30.0% versus the Roche Cobas enzymatic method. These findings demonstrate that the magnitude of apparent POCT bias may depend partly on the comparator methodology [18]. This observation illustrates a broader methodological problem in POCT studies: apparent device bias partly depends on the reference method. A POCT result may agree differently with a Jaffe assay than with an enzymatic assay, even when both central laboratory systems are IDMS traceable. Studies evaluating epoc or other devices should therefore report the comparator methodology, calibration traceability, creatinine range, CKD distribution, and clinically relevant eGFR misclassification rather than relying solely on correlation coefficients.

#### 4.1.5. Radiometer ABL Analyzers

Selected analyzers in the ABL series incorporate creatinine into a broader blood gas and acute-care testing panel. These instruments use an enzymatic amperometric biosensor and can analyze whole blood rapidly while simultaneously reporting blood gases, electrolytes, lactate, and other parameters. Although ABL systems are commonly located near the bedside, they are larger stationary or transportable analyzers rather than truly handheld devices [26,27,28]. Studies of ABL800 and ABL90 platforms have generally reported good precision and clinically acceptable agreement in several settings. The ABL800 (Radiometer Medical ApS, Brønshøj, Denmark) creatinine assay has been evaluated for detecting renal impairment before contrast administration, while analytical evaluation of the ABL90 FLEX PLUS (Radiometer Medical ApS, Brønshøj, Denmark) demonstrated favorable repeatability and agreement with central laboratory enzymatic testing [27,28]. In the systematic evaluation of POCT before contrast-enhanced imaging, ABL800/827 (Radiometer Medical ApS, Brønshøj, Denmark) and i-STAT (Abbott Point of Care Inc., Princeton, NJ, USA) had higher probabilities than StatSensor of correctly identifying patients with eGFR below 30 mL/min/1.73 m^2^ and assigning them to the same eGFR category as the central laboratory [17]. The principal advantages of ABL platforms are analytical automation, integrated quality control, connectivity, and simultaneous measurement of multiple acute-care analytes. Conversely, they require greater capital investment, maintenance, reagent management, and physical infrastructure than handheld meters. They are therefore best considered near-patient laboratory systems, particularly suitable for emergency departments and critical care areas, rather than community or home-testing devices.

#### 4.1.6. Piccolo Xpress

The Piccolo Xpress(Abbott Point of Care Inc., Princeton, NJ, USA) is a compact benchtop chemistry analyzer that uses single-use reagent discs and whole blood, plasma, or serum. Its kidney-oriented panels can provide creatinine and BUN together with other chemistry measurements. Creatinine is measured using coupled enzymatic reactions and photometric detection, with endogenous creatine measured separately and subtracted from the total reaction signal [19]. Independent evaluation has demonstrated acceptable precision, linearity, and agreement for many of the analytes measured by the platform [20]. Its main strength is the ability to obtain a small biochemical panel from one specimen without conventional laboratory infrastructure. This may be useful in outpatient clinics, mobile services, and resource-constrained facilities in which a multifunction chemistry system is preferred over a creatinine-only meter. However, Piccolo Xpress is less portable than handheld systems, requires a larger blood volume, and typically has a longer analysis time than dedicated electrochemical creatinine meters [19,20]. Moreover, acceptable whole-panel performance does not guarantee that creatinine results are interchangeable across all CKD stages. Validation should specifically assess bias at clinically relevant eGFR decision points.

### 4.2. Point-of-Care Urine Albumin-to-Creatinine Ratio Testing

Albuminuria is a core component of CKD detection and risk stratification and should be assessed together with GFR. Machine-read POCT systems can measure urine albumin and urine creatinine and report UACR from a spot urine sample. Examples include the Afinion ACR and Siemens DCA Vantage platforms. Comparative evaluation of 88 spot urine samples found strong correlation with central laboratory measurements for albumin, creatinine, and ACR (R^2^ 0.954–0.991), with total imprecision below 8.7%, although method-specific bias was present [29]. A systematic review of machine-read POC ACR tests supports their potential for albuminuria detection, but performance varies by device and threshold [30]. POCT UACR is therefore best used to facilitate same-visit CKD screening or risk assessment when it improves the care pathway, not as an exemption from confirmation requirements. KDIGO recommends confirming reagent-strip positive albuminuria/proteinuria with quantitative measurement and confirming ACR ≥ 30 mg/g (≥3 mg/mmol) from a random sample with a subsequent first-morning void when appropriate [4]. Biological variability, exercise, infection, menstruation, hydration, and urine-creatinine excretion can affect interpretation. UACR categories remain A1 (<30 mg/g), A2 (30–300 mg/g), and A3 (>300 mg/g), and persistent albuminuria can establish CKD even when eGFR is preserved.

### 4.3. Point-of-Care Cystatin C Testing

Cystatin C is an attractive candidate for next-generation kidney function POCT. It is a low-molecular-weight protein produced by nucleated cells, freely filtered at the glomerulus, and subsequently reabsorbed and catabolized in the proximal tubule. Compared with creatinine, cystatin C is less dependent on skeletal muscle mass and dietary meat intake. Equations combining creatinine and cystatin C generally provide more accurate GFR estimates than equations based on either marker alone [4,15,31,32,33]. Cystatin C is nevertheless not free of non-GFR determinants. Concentrations may be influenced by inflammation, glucocorticoid exposure, thyroid dysfunction, smoking, obesity, and other clinical factors. Its measurement has also historically been limited by assay cost, availability, and imperfect standardization. These issues are important for POCT development because analytical miniaturization cannot correct biological confounding [31,32,33].

#### 4.3.1. Immunosensor Approaches

Because cystatin C is a protein, most emerging POCT methods rely on antibody-based recognition rather than the enzyme-electrode cascades commonly used for creatinine. In an immunosensor, cystatin C binds to a specific capture antibody, and the binding event is converted into an optical, electrochemical, fluorescent, or electrical signal. Sandwich immunoassays may improve specificity by using separate capture and detection antibodies, although they add reagent and fluid-handling complexity [31]. Electrochemical immunosensors are attractive because they can be miniaturized, require relatively simple instrumentation, and may be integrated with disposable electrodes. Reported experimental platforms have used nanomaterials to increase electrode surface area and signal amplification [31]. However, many remain proof-of-concept systems evaluated using buffer, spiked samples, or small clinical datasets. Their progression toward clinical POCT requires assessment of matrix effects, calibration stability, antibody lot variability, sensor fouling, precision, and agreement with internationally standardized laboratory assays.

#### 4.3.2. Microfluidic Platforms

Microfluidic systems manipulate small volumes of fluid through microscale channels, permitting sample separation, reagent mixing, incubation, washing, and signal detection within an integrated cartridge. Their low sample and reagent requirements make them highly compatible with decentralized cystatin C testing. Tomsa et al. developed integrated microfluidic immunoassays with portable readers for serum cystatin C measurement in CKD. The study demonstrated the feasibility of translating cystatin C immunoassays into compact platforms and evaluated performance using clinical serum samples [10]. This represents an important advance beyond purely experimental biosensors because the system integrates assay chemistry, fluid handling, and portable detection. Nevertheless, clinical readiness depends on more than analytical sensitivity. Future platforms must demonstrate traceability to the certified cystatin C reference material, reproducibility across manufacturing lots, robustness under variable temperature and humidity, ease of use with whole blood, and performance across the spectrum of CKD. A clinically useful device should ideally report both cystatin C and cystatin C–based or combined creatinine–cystatin C eGFR.

#### 4.3.3. Future Direction

The most clinically valuable cystatin C POCT platform may not be a stand-alone test. A multiplex cartridge capable of measuring both creatinine and cystatin C from the same sample could simultaneously generate creatinine-based, cystatin C–based, and combined eGFR estimates. Discordance among these estimates could alert clinicians to the presence of non-GFR determinants or unusual body composition. Priorities for future research include whole-blood testing without extensive sample preparation; calibration traceable to international cystatin C reference standards; clinically appropriate precision around CKD staging thresholds; validation in older adults and in patients with sarcopenia, advanced CKD, obesity, thyroid disease, or glucocorticoid exposure; direct comparison with measured GFR; and prospective studies demonstrating that rapid cystatin C testing changes clinical management or improves patient outcomes. At present, cystatin C POCT should therefore be regarded as an emerging technology rather than an established replacement for conventional laboratory testing.

## 5. Research Horizon: Kidney Injury, Stress, and Molecular Biomarkers

Beyond filtration and albuminuria, several biomarkers may characterize tubular injury, cellular stress, or molecular phenotype. These analytes should not be interpreted as direct measures of GFR, and for stable CKD most lack validated decision thresholds or evidence that POCT-guided management improves outcomes. Accordingly, this section is presented as a research horizon. NGAL and [TIMP-2]·[IGFBP7] have the greatest near-patient maturity, but their established evidence base is predominantly AKI rather than stable CKD. L-FABP has a semiquantitative urine POC kit in selected markets, whereas KIM-1 and urinary microRNA have been evaluated using proof-of-concept electrochemical sensing platforms, including studies using urine or other biological samples. These technologies nevertheless remain research-stage and have not been validated for routine CKD point-of-care use [34,35]. Extracellular-vesicle and other molecular platforms remain at the research stage. Table 2 therefore reports readiness conservatively and separates analytical/technology maturity from clinical interpretability in CKD.

### 5.1. Neutrophil Gelatinase-Associated Lipocalin

NGAL is a small protein expressed by injured tubular epithelial cells and may rise in plasma and urine before substantial creatinine change during acute tubular injury. Commercial rapid immunoassays and near-patient platforms have made NGAL one of the most clinically advanced injury biomarkers [36,37]. In CKD, however, interpretation is complicated by reduced renal clearance, chronic tubular damage, systemic inflammation, and neutrophil production. Plasma and urinary NGAL concentrations can correlate with lower eGFR and greater albuminuria even in the absence of superimposed AKI. Thus, elevated NGAL in CKD is not specific for acute injury. Potential POCT applications include detection of acute-on-chronic kidney injury, peri-procedural risk assessment, and monitoring of nephrotoxic exposure, but CKD-specific thresholds and serial-change criteria require validation.

### 5.2. Kidney Injury Molecule-1

KIM-1 is a transmembrane protein that is minimally expressed in healthy kidneys but markedly upregulated in dedifferentiated proximal tubular cells after injury. Its extracellular domain is shed into urine, making urinary KIM-1 an attractive non-invasive marker of proximal tubular damage [38]. KIM-1 has been investigated in AKI, diabetic kidney disease, toxic nephropathy, and CKD progression. Immunoassays and experimental electrochemical sensors could potentially be adapted to portable platforms. Major barriers include the absence of universally accepted clinical cutoffs, variation with underlying CKD etiology, and limited evidence that KIM-1-guided decisions improve outcomes [38]. Its future role may be strongest in multiplex panels rather than as an isolated marker.

### 5.3. Liver-Type Fatty Acid-Binding Protein

L-FABP is expressed in proximal tubular cells and participates in intracellular fatty-acid transport. Urinary L-FABP increases in response to tubular hypoxia and oxidative stress and has been studied in AKI and progressive CKD, particularly diabetic kidney disease [39]. Rapid immunochromatographic and immunoassay-based methods are technically feasible, but widespread POCT adoption remains limited. Urinary concentration may be affected by urine dilution, and results may require normalization to urine creatinine, adding analytical complexity. Future studies should determine whether serial L-FABP measurement provides information beyond eGFR and UACR in CKD.

### 5.4. β2-Microglobulin

β2-Microglobulin is a low-molecular-weight component of major histocompatibility complex class I molecules. It is freely filtered by the glomerulus and almost completely reabsorbed and catabolized by proximal tubular cells. Serum concentrations therefore reflect filtration but are also influenced by inflammation, infection, malignancy, and immune activation. Urinary β2-microglobulin may indicate impaired proximal tubular reabsorption [40]. From a POCT perspective, β2-microglobulin has the advantage of being measurable by immunoassay and may provide complementary filtration information when creatinine is unreliable. However, its extrarenal determinants and instability in acidic urine limit its use as a stand-alone kidney function marker. Clinical POCT systems remain underdeveloped compared with creatinine platforms.

### 5.5. β-Trace Protein

β-Trace protein, also known as prostaglandin D synthase, is a low-molecular-weight glycoprotein that has been evaluated as an alternative endogenous filtration marker. Serum β-trace protein is less dependent on muscle mass than creatinine and can be incorporated into GFR-estimating equations, including equations combining multiple filtration markers [41,42]. Potential limitations include effects of corticosteroids, thyroid function, inflammation, and differences in assay calibration. Although immunosensor miniaturization is technically plausible, β-trace protein has not yet progressed to routine kidney POCT. Its future value may lie in multi-marker panels designed to reduce the influence of non-GFR determinants affecting any single biomarker.

### 5.6. Uromodulin

Uromodulin is produced predominantly by epithelial cells in the thick ascending limb and early distal tubule. Unlike injury markers that increase after cellular damage, circulating and urinary uromodulin may reflect tubular mass, integrity, and functional reserve. Lower serum or urinary concentrations have been associated with reduced kidney function and adverse CKD outcomes [43,44]. Uromodulin may therefore complement glomerular filtration markers by providing information about tubular health. However, assay standardization, biological variability, urinary normalization, and clinically actionable thresholds remain unresolved. POCT development is currently at an early stage.

### 5.7. TIMP-2 and IGFBP7

TIMP-2 and IGFBP7 are urinary cell-cycle arrest biomarkers released during tubular stress. The product of their concentrations, [TIMP-2]·[IGFBP7], is used clinically to identify critically ill patients at increased risk of developing moderate-to-severe AKI [45,46]. These biomarkers represent kidney stress, not GFR, and their established clinical context is AKI risk prediction rather than CKD staging. In CKD, chronically altered baseline concentrations could complicate interpretation. A possible future role is the identification of subclinical acute stress in patients with CKD before overt creatinine elevation for example, during sepsis, surgery, or nephrotoxic treatment. CKD-specific validation and demonstration of management benefit are needed before routine integration into chronic kidney care.

### 5.8. Urinary Extracellular Vesicles and Exosomes

Urinary extracellular vesicles, including exosomes, are released by cells throughout the nephron and contain proteins, lipids, messenger RNA, and microRNA reflecting their cell of origin. They may provide a molecular snapshot of ongoing glomerular and tubular processes and have been investigated in diabetic kidney disease, IgA nephropathy, polycystic kidney disease, transplant injury, and other kidney disorders [47,48]. Their potential advantages include non-invasive sampling and the ability to capture disease-specific molecular information. However, current workflows require vesicle isolation, purification, molecular extraction, and specialized analysis. Preanalytical variation related to urine collection, storage, concentration, and isolation method remains substantial. Microfluidic capture and lab-on-chip analysis may ultimately enable POCT, but urinary exosome testing currently remains a research technology.

### 5.9. MicroRNA

MicroRNAs are short non-coding RNAs that regulate post-transcriptional gene expression. Disease-associated microRNA patterns have been detected in blood, urine, and urinary extracellular vesicles and may differentiate CKD etiologies or predict progression [49,50]. For POCT, nucleic-acid amplification, electrochemical detection, and nanomaterial-assisted sensors may eventually permit rapid multiplexed analysis. Nevertheless, low analyte abundance, normalization strategies, extraction requirements, biological heterogeneity, and lack of standardized reference intervals currently preclude routine use. Rather than functioning as general filtration markers, miRNA panels are more likely to support molecular phenotyping and precision diagnosis.

## 6. Evidence for Clinical Applications of Point-of-Care Kidney Assessment

**Figure 1:**
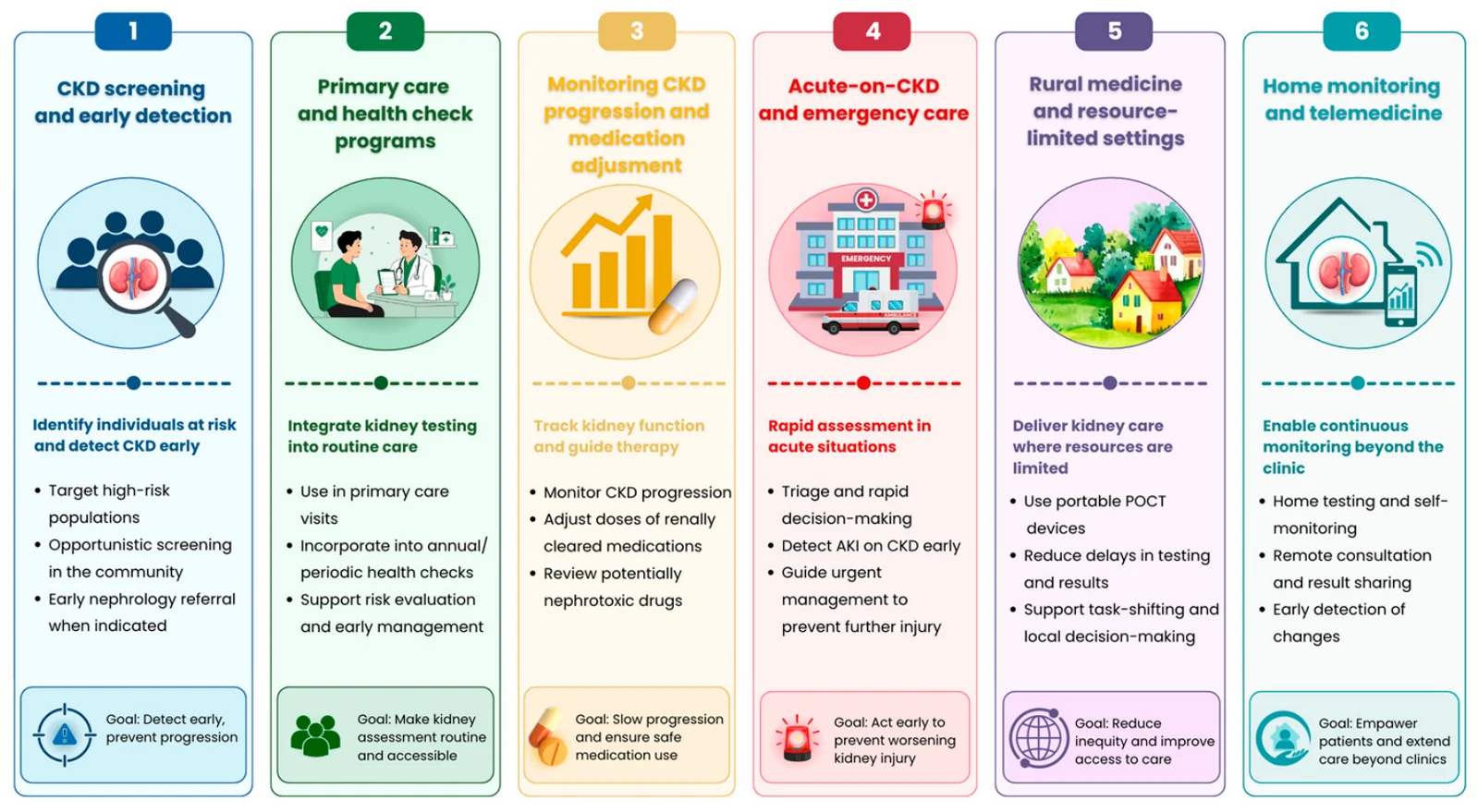
Evidence-informed applications of point-of-care kidney assessment across CKD care. Current uses should be distinguished from investigational future applications, such as home CKD monitoring and telemedicine-integrated self-testing.

Although analytical performance is fundamental, the ultimate value of point-of-care kidney function testing lies in its ability to improve clinical decision-making. Rapid measurement of kidney function may shorten diagnostic pathways, facilitate timely therapeutic interventions, reduce unnecessary delays, and improve access to kidney care, particularly in settings where conventional laboratory infrastructure is unavailable or laboratory turnaround time is prolonged. Current clinical applications extend from community-based CKD screening to acute hospital care and telemedicine-supported disease management [4,16]. The major clinical applications of point-of-care kidney function testing across the CKD care continuum are summarized in Figure 1.

### 6.1. CKD Screening and Early Detection

CKD remains substantially underdiagnosed worldwide, with many patients remaining asymptomatic until advanced disease develops. Consequently, early detection relies on laboratory assessment of kidney function and albuminuria among high-risk individuals, including those with diabetes, hypertension, obesity, and cardiovascular disease [4]. Current KDIGO guidelines recommend targeted CKD screening using serum creatinine-derived eGFR together with urine albumin measurement rather than population-wide screening [4]. Portable creatinine POCT devices allow immediate estimation of eGFR during a single patient encounter, thereby reducing loss to follow-up and enabling prompt counseling, referral, or initiation of kidney-protective therapy [16,17].

Community-based screening studies have demonstrated that handheld creatinine analyzers are feasible for identifying previously unrecognized CKD in rural and underserved populations, although abnormal results should always be confirmed using standardized laboratory assays because CKD is defined by abnormalities of kidney structure or function present for at least three months; persistent albuminuria may establish CKD even when eGFR is preserved [23]. The combination of rapid creatinine measurement with portable urine albumin testing may therefore represent an effective strategy for decentralized CKD screening in primary healthcare systems.

### 6.2. Primary Care and Health Check Programs

Primary care is a logical setting for kidney POCT because many patients at risk for CKD are assessed outside nephrology clinics [51]. Same-visit creatinine/eGFR and UACR results may reduce pathway delays and support risk communication or referral decisions when conventional laboratory access is limited. However, evidence that POCT itself increases guideline-concordant annual testing or improves long-term kidney outcomes remains limited; these should be considered potential workflow benefits rather than established patient-level clinical utility. Abnormal results should be confirmed according to CKD diagnostic criteria and local quality-assurance procedures [4,17,30].

### 6.3. Monitoring CKD Progression and Medication Adjustment

Serial kidney-function assessment is required for many CKD therapies, but this does not establish an evidence-based indication for POCT. Creatinine may need to be checked after initiation or dose adjustment of renin–angiotensin system inhibitors, diuretics, selected antimicrobials, calcineurin inhibitors, or other drugs, and kidney function is routinely monitored during SGLT2 inhibitor therapy [4,52,53,54,55]. POCT can be considered when a same-visit result would materially alter the clinical pathway, but the added benefit over timely central-laboratory testing has not been established for most medication-monitoring scenarios. For drug dosing, clinicians should use the renal-function measure specified for the individual medication and should not assume that indexed POCT-derived eGFR is interchangeable with absolute GFR or creatinine clearance.

### 6.4. Acute-on-Chronic Kidney Disease and Emergency Care

Patients with CKD may present with acute illness and superimposed AKI, where rapid creatinine measurement can be operationally useful. However, creatinine-based eGFR equations assume approximate steady state and should not be interpreted as a precise measure of GFR during rapidly evolving AKI. In this setting, the serial creatinine concentration, urine output, prior baseline values, clinical context, and—when relevant—kinetic approaches are more informative than an automatically displayed eGFR. Near-patient creatinine can shorten turnaround time, but evidence for improved patient-level outcomes remains limited [17,56].

### 6.5. Rural Medicine and Resource-Limited Settings

Limited laboratory infrastructure remains a major obstacle to CKD diagnosis in low- and middle-income countries [23]. Portable creatinine analyzers require only small blood volumes and minimal infrastructure, making them suitable for outreach clinics, mobile health services, humanitarian missions, and geographically isolated communities [16]. These technologies may substantially reduce delays associated with specimen transportation while improving access to kidney function assessment in underserved populations. Successful implementation nevertheless requires robust quality assurance, operator training, external quality assessment, and sustainable supply chains [13,16].

### 6.6. Home Monitoring and Telemedicine

Telemedicine creates a potential future pathway for decentralized CKD monitoring, but home creatinine and cystatin C testing remain investigational. Prototype microfluidic cystatin C systems demonstrate analytical feasibility in clinical samples [10], yet prospective evidence is still needed for user performance, quality control, secure data transfer, clinical action thresholds, cost-effectiveness, and patient-centered outcomes. Accordingly, home kidney POCT is discussed as a future application rather than an established component of current CKD care.

## 7. Comparative Analytical Performance, Implementation, and Quality Assurance

For kidney POCT, four levels of evidence should be distinguished: analytical validity (bias, imprecision, interference, traceability), clinical validity (ability to classify clinically relevant kidney states), workflow utility (turnaround time or pathway efficiency), and patient-level clinical utility (improved safety, outcomes, adherence, or cost-effectiveness). The literature is strongest for analytical validity and selected workflow outcomes; patient-level utility remains much less certain. Consequently, rapid turnaround alone should not be interpreted as proof of clinical benefit. The principal analytical and implementation issues are summarized in Table 3.

### 7.1. Accuracy, Precision, and Clinical Agreement

Analytical accuracy describes the closeness of a measured value to the true value, whereas precision reflects the reproducibility of repeated measurements under identical conditions. Both characteristics are essential because an assay may demonstrate excellent precision while remaining systematically biased, or conversely, produce unbiased average results but poor repeatability [13]. Method-comparison studies should therefore evaluate both systematic and proportional bias using Bland–Altman analysis or regression techniques that account for measurement error in both methods, such as Passing–Bablok or Deming regression, rather than relying solely on correlation coefficients [13,27,57]. High correlation may simply reflect a wide distribution of creatinine concentrations and should not be interpreted as evidence of analytical interchangeability [27,58]. From a clinical perspective, agreement at relevant eGFR thresholds may be more informative than numerical agreement alone. Misclassification around thresholds of 30, 45, or 60 mL/min/1.73 m^2^ may influence CKD staging, contrast administration, medication dosing, and nephrology referral despite relatively small absolute differences in creatinine concentration [17,22].

### 7.2. Calibration, Reference Methods, and Standardization

Reliable POC testing depends on calibration traceable to internationally accepted reference measurement systems. The global standardization of serum creatinine following IDMS traceability has substantially improved comparability between laboratory assays and enabled more consistent application of creatinine-based eGFR equations [13,18]. Nevertheless, calibration differences may still exist among POC devices because manufacturers employ different analytical methodologies, sensor technologies, calibration procedures, and quality-control systems. Consequently, analytical agreement should always be assessed against an IDMS-traceable laboratory assay rather than another POC platform [17,24]. Similar challenges remain for cystatin C. International reference material (ERM-DA471/IFCC) has improved assay harmonization; however, complete standardization has not yet been achieved across all commercial platforms, particularly for emerging POC technologies [15,33]. These issues are especially relevant because even modest analytical bias may propagate into clinically meaningful errors in eGFR estimation.

### 7.3. Inter-Device Variability

Analytical performance cannot be generalized across all POC platforms. Differences in biosensor design, enzymatic reactions, detection technology, sample handling, and internal calibration may produce device-specific bias even when instruments are traceable to the same reference method [22,24]. Comparative studies evaluating commercially available creatinine POCT systems have demonstrated that i-STAT and selected Radiometer ABL analyzers generally show closer agreement with central laboratory measurements than some handheld electrochemical systems, whereas creatinine bias may be greater with certain platforms at higher creatinine concentrations [17,22,24]. These observations should not be interpreted as universal rankings because analytical performance varies according to patient population, specimen type, comparator method, and device generation. Accordingly, institutions introducing POC kidney function testing should perform local verification studies and ongoing quality assurance rather than assuming equivalent performance across manufacturers.

### 7.4. Biological and Analytical Interference

Like conventional laboratory assays, POC creatinine measurements remain susceptible to biological and analytical interference. The magnitude and direction of interference depend on the analytical principle used and should not be generalized across different devices [16,27]. Hematocrit represents one of the most important potential sources of analytical variation for whole-blood POC systems. Severe anemia or polycythemia may alter sample conductivity, plasma fraction, or electrochemical signal generation, potentially affecting measured creatinine concentrations in selected devices [21,24]. Protein abnormalities may also influence assay performance. Severe hypoproteinemia may alter plasma water fraction and indirectly affect certain measurement systems, whereas hyperbilirubinemia, hemolysis, lipemia, and endogenous chromogens have long been recognized as important analytical interferents, particularly for Jaffe-based creatinine assays [18,27].

Drug-related interference should likewise be considered when interpreting unexpected creatinine results. Certain medications, including cephalosporins, dopamine, dobutamine, ascorbic acid, and acetaminophen, may interfere analytically with selected creatinine methodologies, whereas trimethoprim and cimetidine increase serum creatinine by inhibiting tubular secretion without reducing true GFR [16,27]. Clinicians should therefore distinguish analytical interference from physiological changes in creatinine metabolism or renal handling.

### 7.5. Challenges at Extreme Kidney Function

The analytical requirements for POC kidney function testing become increasingly demanding at the extremes of kidney function. At very low creatinine concentrations, relatively small absolute analytical errors may produce disproportionately large differences in eGFR, potentially altering CKD classification in individuals with preserved kidney function [4]. Conversely, at advanced CKD or kidney failure, high creatinine concentrations may exceed the optimal analytical range of certain devices, and proportional bias becomes more clinically relevant because medication dosing, dialysis planning, and referral decisions often depend on relatively small changes in measured creatinine [22,24]. For these reasons, unexpected POC results particularly when inconsistent with the clinical presentation or obtained near important therapeutic thresholds should be confirmed using standardized central laboratory testing before major management decisions are made.

## 8. Future Perspectives

Rapid advances in biosensor technology, digital health, and decentralized healthcare delivery are expected to expand the role of point-of-care kidney function testing beyond isolated biochemical measurements. Future POC platforms may increasingly function as components of integrated healthcare systems that combine biochemical testing, electronic health records, cloud-based data management, and clinical decision support. However, although many of these technologies are technically feasible, evidence demonstrating improvements in patient outcomes remains limited, and further prospective validation is required before widespread clinical implementation [4,59].

### 8.1. Artificial Intelligence and Clinical Decision Support

AI has the potential to enhance the clinical utility of kidney POC testing by assisting clinicians with interpretation rather than replacing biochemical measurements. Machine-learning algorithms can integrate serial creatinine values, cystatin C, albuminuria, demographic characteristics, medication history, and longitudinal electronic health record data to predict CKD progression, identify patients at high risk of acute kidney injury, and support individualized clinical decision-making [52,59,60]. Rather than generating independent diagnoses, future AI-assisted POC systems may automatically identify unexpected changes in kidney function, recognize discordance between biomarkers, and recommend confirmatory laboratory testing or nephrology referral when clinically appropriate. Nevertheless, prospective interventional studies demonstrating that AI-assisted POC testing improves patient outcomes remain scarce, and clinical implementation should therefore proceed cautiously [59,60].

### 8.2. Digital Health and Cloud-Based Connectivity

Modern POC devices increasingly incorporate digital connectivity, allowing laboratory results to be transferred directly to electronic health records or cloud-based healthcare platforms. Such integration may reduce transcription errors, facilitate longitudinal assessment of kidney function, and improve communication between primary care physicians and nephrologists [16,60]. Cloud-based data management may also support remote review of kidney function trends, enabling specialist consultation for patients in geographically isolated areas and facilitating multidisciplinary CKD care. However, successful implementation will require robust cybersecurity, interoperability between healthcare systems, protection of patient privacy, and appropriate regulatory oversight [60].

### 8.3. Wearable and Non-Invasive Biosensors

One of the major limitations of current kidney function assessment is the requirement for blood sampling. Consequently, considerable research has focused on wearable and minimally invasive biosensors capable of detecting biochemical analytes in alternative biological fluids, particularly sweat. Experimental wearable electrochemical sensors have demonstrated promising analytical performance for monitoring glucose, electrolytes, lactate, and other metabolites, illustrating the feasibility of continuous biochemical sensing in ambulatory settings [61,62]. However, validated wearable sensors capable of accurately measuring established kidney filtration markers such as creatinine or cystatin C remain unavailable for routine clinical practice.

Saliva has also been investigated as a non-invasive source of kidney biomarkers because creatinine, urea, uric acid, phosphate, and several inflammatory mediators can be detected in oral fluid. Nevertheless, salivary biomarker concentrations are influenced by salivary flow rate, hydration status, oral disease, and assay methodology, and current evidence is insufficient to support salivary testing as a replacement for conventional blood-based assessment of kidney function [63]. Accordingly, wearable and saliva-based kidney monitoring should currently be regarded as promising research technologies rather than clinically validated alternatives to conventional laboratory testing.

### 8.4. Telemedicine and Remote Kidney Care

Telemedicine has become an increasingly important component of CKD management, particularly for patients living in rural or underserved regions. Future POC kidney function testing may complement virtual nephrology consultations by allowing patients to perform creatinine testing before scheduled remote appointments, thereby facilitating medication adjustment, monitoring of disease progression, and early identification of clinically significant deterioration [60]. The combination of home-based POC testing, cloud-based data transmission, and telemedicine may improve access to specialist kidney care while reducing unnecessary hospital visits. However, implementation will require standardized quality assurance procedures, user-friendly sampling methods, secure electronic data transfer, and demonstration of cost-effectiveness in prospective clinical studies.

### 8.5. Toward Integrated Precision Kidney Care

**Figure 2:**
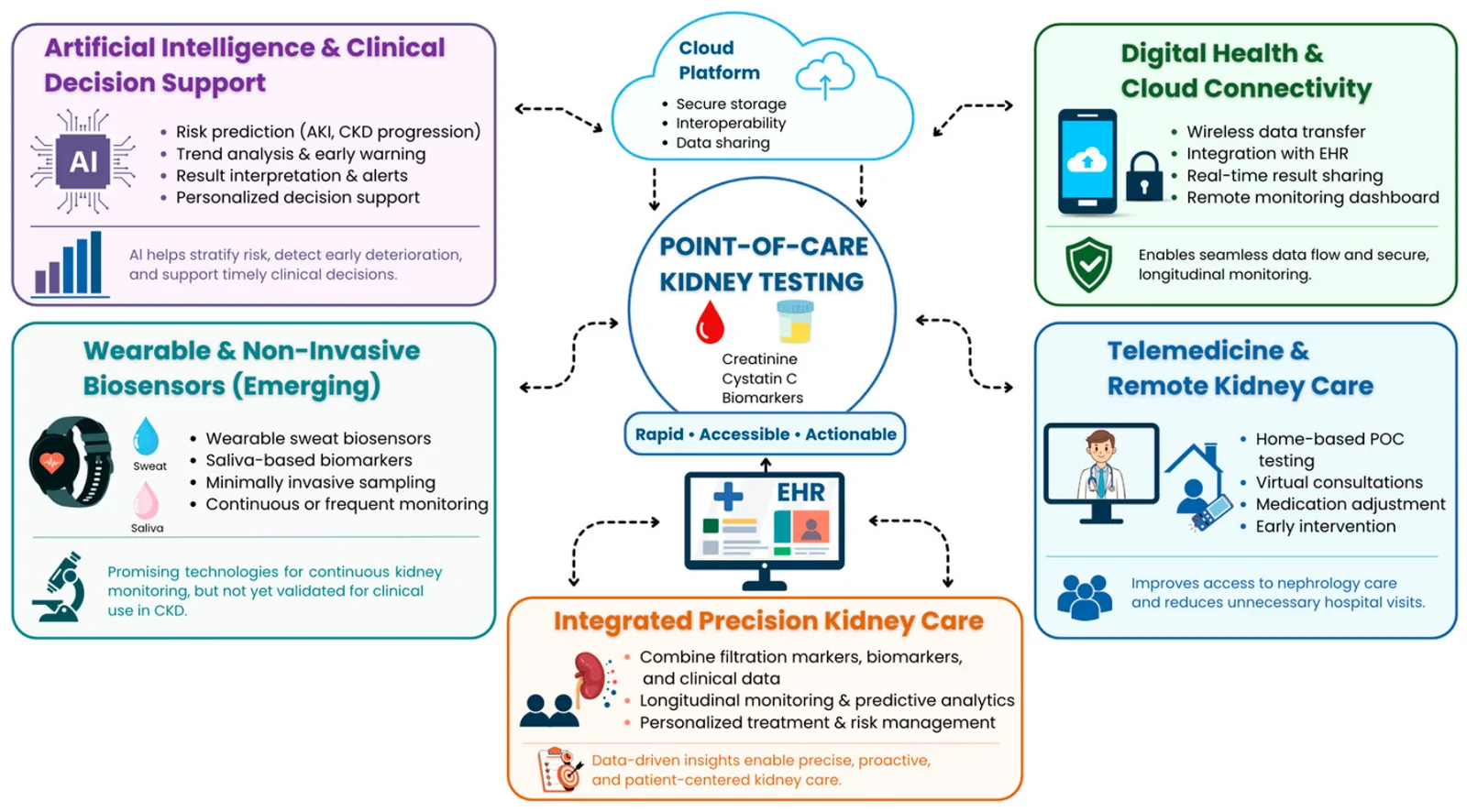
Conceptual research horizon for point-of-care kidney assessment, including multiplex biomarkers, digital connectivity, artificial intelligence, and remote care. Arrows indicate interconnections and bidirectional data flow among the components. These elements represent future directions rather than established clinical utility.

Future kidney POC platforms are unlikely to rely on a single biomarker. Instead, the next generation of diagnostic systems will probably integrate complementary filtration markers, such as creatinine and cystatin C, with longitudinal clinical information and digital decision-support tools. Such multidimensional approaches may improve recognition of clinically meaningful changes in kidney function while reducing reliance on isolated creatinine measurements [60]. Nevertheless, future technologies should ultimately be evaluated according to their ability to improve patient-centered outcomes, healthcare accessibility, and cost-effectiveness rather than analytical innovation alone. Until such evidence becomes available, standardized laboratory testing should remain the reference standard for the diagnosis and long-term monitoring of chronic kidney disease. The potential integration of these emerging technologies into a connected ecosystem for kidney function testing is illustrated in Figure 2.

### 8.6. Research Priorities

Priority research areas include: (1) prospective studies testing whether POCT-guided pathways improve patient-centered outcomes rather than turnaround time alone; (2) cost-effectiveness and budget-impact analyses across primary care, imaging, emergency, rural, and low-resource settings; (3) implementation-science studies addressing operator competency, external quality assessment, connectivity, and sustainability; (4) head-to-head validation of multiplex platforms combining creatinine, cystatin C, and albuminuria, with injury biomarkers added only when they provide incremental clinical value; (5) validation of home and self-testing under real-world user conditions; (6) standardization of reporting around bias, imprecision, clinically relevant misclassification, and assay traceability; and (7) trials evaluating whether digital decision support or AI coupled to POCT changes management safely and improves kidney, cardiovascular, quality-of-life, or healthcare-utilization outcomes.

## 9. Conclusions

POCT can provide rapid kidney assessment and may improve workflow or access in selected settings, but the maturity of evidence varies substantially by analyte and application. The strongest current evidence concerns creatinine POCT for selected decentralized and pre-imaging workflows and machine-read UACR testing for albuminuria detection with appropriate confirmation. These technologies should complement, not automatically replace, standardized laboratory testing, particularly for longitudinal CKD monitoring or results near major clinical decision thresholds.

Cystatin C POCT, home monitoring, AI-assisted interpretation, and injury or molecular biomarker platforms remain investigational or incompletely validated for routine CKD care. Future adoption should be driven by demonstrated analytical quality, clinical validity, workflow benefit, cost-effectiveness, and—most importantly—patient-centered clinical utility rather than technical feasibility alone.

## Figures and Tables

**Table 1:** Quantitative and practical characteristics of representative commercially available point-of-care creatinine platforms. Specifications are configuration- and jurisdiction-dependent; manufacturer claims should be verified locally before implementation.

Platform	Sample/Volume	Range/Time	Principle	eGFR Reporting	Comparative Evidence	Configuration/Availability Caveat
Nova StatSensor	Whole blood; 1.2 µL [21]	0.30–12.0 mg/dL; ~30 s [21]	Enzymatic amperometry	Yes; calculated. CKD-EPI/MDRD available	Proportional bias; wider variability than i-STAT/ABL [17,22,23]	eGFR options and commercial claims vary by model/jurisdiction
Abbott i-STAT CREA	Arterial/capillary/venous whole blood; 65 µL [24]	0.2–20.0 mg/dL; ~2 min [24]	Enzymatic amperometry	Yes; calculated from creatinine; software/equation dependent	Generally favorable agreement; better eGFR < 30 classification than StatSensor [17,23,24]	eGFR display and cartridge/regulatory status vary by software/country
Siemens epoc	Whole blood; card-specific volume [25]	0.30–15.00 mg/dL; minutes [25]	Enzymatic amperometry	Yes; calculated. MDRD/CKD-EPI options	Smaller evidence base; method-dependent bias reported [17]	Software and regulatory configuration vary
Radiometer ABL90	Whole blood; ABL90 Crea/Urea ~65 µL [28]	Model-specific; ABL90 ~35 s [28]	Enzymatic amperometry	Yes; calculated; model/configuration dependent	Selected systems show favorable agreement and eGFR < 30 classification [17,27,28]	Near-patient rather than handheld; model/software dependent
Piccolo Xpress	Whole blood/plasma/serum; disc-specific [19]	Panel-specific; ~12 min [19,20]	Coupled enzymatic photometry	Yes; calculated; creatinine-containing panel dependent	Limited comparative creatinine evidence; acceptable panel performance [17,20]	Disc menu and availability vary by market

**Table 2:** Emerging biomarkers for point-of-care kidney function assessment: biological roles, clinical applications, and current development status.

Biomarker	Predominant Biological Domain	Potential Role	POCT Maturity	Major Limitation in CKD
Cystatin C	Glomerular filtration	Alternative or confirmatory eGFR	Prototype microfluidic clinical-sample validation; not routine POCT	Non-GFR determinants and assay cost
NGAL	Tubular injury/inflammation	Acute-on-CKD injury detection	Commercial rapid/near-patient assays; mainly AKI evidence	Elevated in CKD and systemic inflammation
KIM-1	Proximal tubular injury	Active tubular damage	Proof-of-concept electrochemical sensors; not routine clinical POCT	No standardized clinical thresholds
L-FABP	Tubular hypoxia/oxidative stress	Early tubular stress and prognosis	Semiquantitative urine POC kit available in selected markets; CKD interpretation not standardized	Urine dilution and disease heterogeneity
β2-Microglobulin	Filtration/tubular reabsorption	Alternative filtration and tubular marker	Laboratory immunoassay feasible; limited dedicated kidney POCT evidence	Inflammation, malignancy, immune activation
β-Trace protein	Glomerular filtration	Alternative endogenous filtration marker	Research/early development	Limited standardization and clinical availability
Uromodulin	Tubular integrity and nephron mass	Tubular reserve and CKD prognosis	Research stage	Biological variability and uncertain thresholds
[TIMP-2]·[IGFBP7]	Tubular cellular stress	AKI risk in patients with CKD	Established near-patient AKI-risk platform; not CKD staging	Not a measure of GFR; limited stable-CKD role
Urinary exosomes	Molecular and cellular phenotype	Disease classification and monitoring	Research stage; complex preanalytics	Complex isolation and preanalytics
MicroRNA	Gene-regulatory signature	Molecular phenotyping and progression	Proof-of-concept electrochemical detection in small clinical samples; research stage	Extraction, normalization, and validation

**Table 3:** Key analytical challenges of point-of-care kidney function testing and their clinical implications.

Analytical Challenge	Key Analytical Issue	Potential Clinical Impact	Practical Consideration
Accuracy, precision, and clinical agreement	Bias and imprecision; high correlation does not ensure agreement	Misclassification of kidney function or apparent changes unrelated to biological variation	Assess bias, precision, and agreement at clinically relevant eGFR thresholds
Calibration, reference methods, and standardization	Differences in calibration, traceability, and comparator methods	Reduced comparability between POCT and central laboratory results	Use IDMS-traceable creatinine assays and clearly report the reference method
Inter-device variability	Device-specific differences in analytical methods, calibration, and software	Performance may not be generalizable across platforms	Perform local verification and ongoing quality assurance
Biological and analytical interference	Hematocrit, bilirubin, hemolysis, lipemia, proteins, and selected medications may affect results	Over- or underestimation of creatinine	Consider assay-specific interference and confirm unexpected results
Challenges at extreme kidney function	Creatinine error may disproportionately affect eGFR, particularly near clinical thresholds	Incorrect CKD classification or inappropriate clinical decisions	Confirm unexpected or threshold-adjacent results with standardized laboratory testing

## Data Availability

No new data were created or analyzed in this study. Data sharing is not applicable to this article.

## Abbreviations

The following abbreviations are used in this manuscript:

ACEI	Angiotensin-converting enzyme inhibitor
AI	Artificial intelligence
AKI	Acute kidney injury
ARB	Angiotensin receptor blocker
BUN	Blood urea nitrogen
CKD	Chronic kidney disease
CKD-EPI	Chronic Kidney Disease Epidemiology Collaboration
eGFR	Estimated glomerular filtration rate
EHR	Electronic health record
ERM-DA471/IFCC	European Reference Material DA471/International Federation of Clinical Chemistry and Laboratory Medicine
GFR	Glomerular filtration rate
IDMS	Isotope dilution mass spectrometry
IGFBP7	Insulin-like growth factor-binding protein 7
KDIGO	Kidney Disease: Improving Global Outcomes
KIM-1	Kidney injury molecule-1
L-FABP	Liver-type fatty acid-binding protein
NGAL	Neutrophil gelatinase-associated lipocalin
POCT	Point-of-care testing
QC	Quality control
SGLT2	Sodium-glucose cotransporter 2
TIMP-2	Tissue inhibitor of metalloproteinases-2
UACR	Urine albumin-to-creatinine ratio
